# A multicriteria adaptive opportunistic treecast routing protocol for multimedia dissemination in vehicle-to-vehicle telescreen

**DOI:** 10.1371/journal.pone.0205081

**Published:** 2018-10-26

**Authors:** Ghulam Sarwar, Sungchang Lee

**Affiliations:** School of Electronics and Information Engineering, Korea Aerospace University, Deogyang-gu, Goyang-si, Gyeonggi-do, Korea; Northeastern University, CHINA

## Abstract

This paper presents vehicle-to-vehicle telescreen (VVT) and a multicast scheme to disseminate digital signage multimedia services to vehicular ad hoc networks (VANETs). Multimedia dissemination in VANETs is challenging because of the high packet losses (PLs), delays and longer disconnection times, which degrade the network quality of service (QoS) and user quality of experience (QoE). To reduce the PLs and delays, most existing multimedia multicast schemes in VANETs primarily select routes based on longer route expiration times (RET) or lowest path delays. The RET-based schemes suffer less from PLs when there are fewer active multicastings in the network. When the number of active multicastings increases, delay-based schemes suffer less from PLs comparatively. This tradeoff implies to design an adaptive mechanism by mutually complementing the RET-based and delay-based schemes to reduce PLs and delays. In this paper, we propose a multicriteria adaptive opportunistic treecast routing protocol (MAOTRP), which adapts the route selection mechanism according to active multicastings for efficient multimedia dissemination in VVT. The MAOTRP adjusts the weights of route selection parameters, including RET and delays, by considering their contribution in improving packet delivery ratio. MAOTRP extends a tree-based multicast protocol to provide robustness through alternate routes for link failures to reduce PLs. Through several experimental evaluations, we show that the proposed dissemination scheme improves QoS and QoE, and reduces the average disconnection time.

## 1 Introduction

The convergence of broadband access networks and vehicular ad hoc networks (VANETs) provides a platform to share and access multimedia contents such as safety and infotainment services. Vehicle-to-vehicle telescreen (VVT) extends digital signage (DS) infotainment and advertisement services to drivers and passengers on the road. According to forecasts, the use of real-time video services in VANETs will double in the next few years [1, 2]. However, enabling a scalable VVT service is very challenging due to the stringent delay requirements of real-time video services, the limited transmission range, and the dynamic number of active multicastings and VANET topologies. VVT uses IEEE 802.11p-based limited-transmission range communication technology for vehicle-to-vehicle (V2V) and vehicle-to-infrastructure (V2I and I2V) communication.

**Fig 1:**
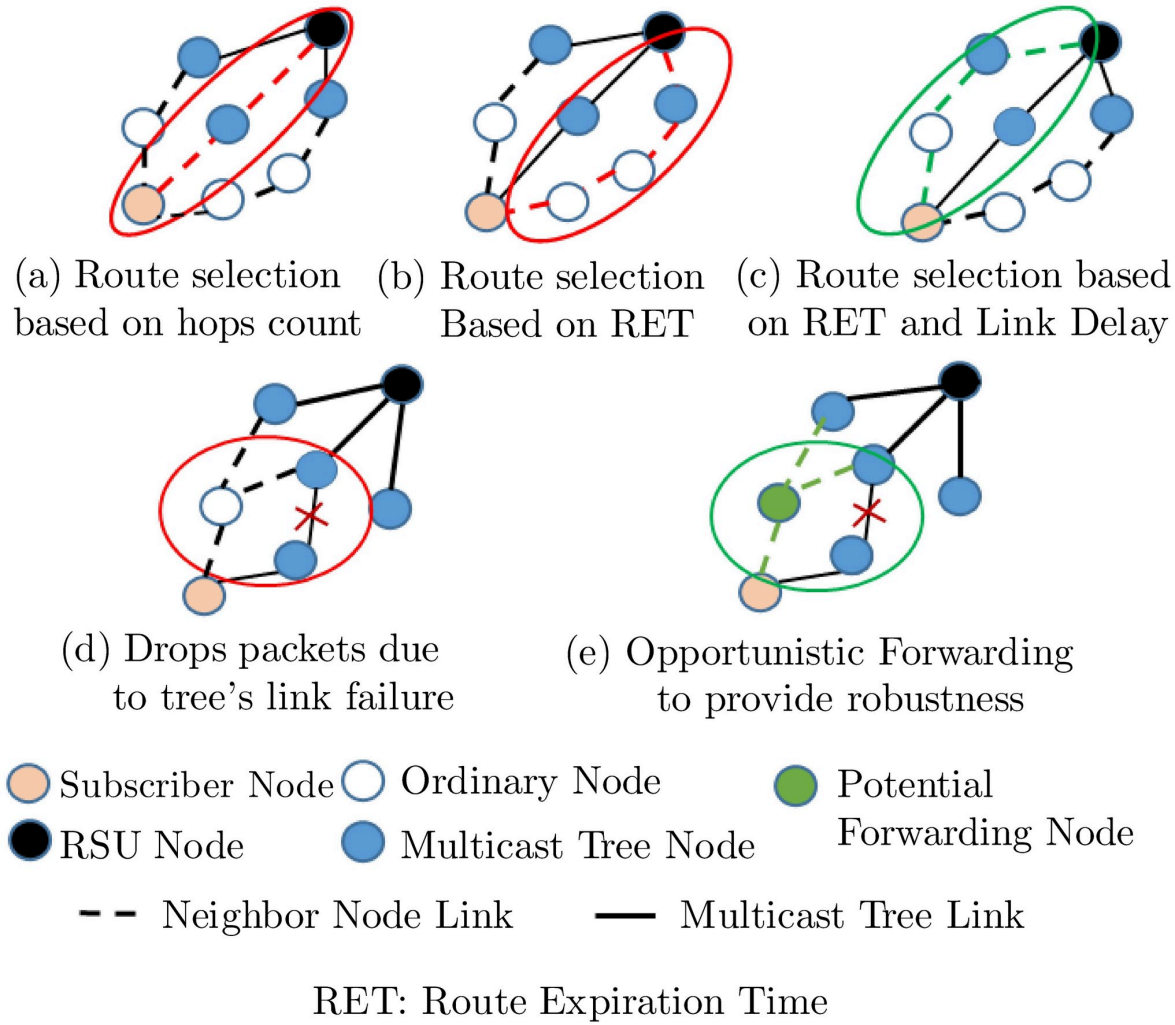
Overview of the multicast route selection criteria and opportunistic routes.

VVT service provisioning process includes service’s advertisements, subscriptions, and dissemination, which can be efficiently supported using an appropriate multicast protocol. There are two basic categories of multicast protocols in VANETs − tree-based and mesh-based multicast routing protocols. Tree-based multicast schemes select routes based on the number of hops [3], or the lifetime [4], as depicted in Fig 1a and 1b. These schemes are delay efficient at the expense of low robustness because they do not consider alternate routes and drop packets due to disconnections, collisions or low signal-to-noise ratios (SNRs) (Fig 1d). On the other hand, mesh-based multicast schemes consider alternate routes to provide robustness at the expense of higher maintenance overheads [5]. In mesh-based schemes, nodes share routing tables with neighbors, but maintaining accurate information in dynamic topologies increases the network congestion and packet collisions.

There are intrinsic trade-offs between the selection of the shortest delay and the least disconnected route for multi-hop multimedia dissemination. Delay-based route selection schemes (DBRSSes) choose paths offering shorter latency. However, the selected routes may suffer from frequent disconnections in dynamic VANET environments. In contrast, lifetime-based route selection schemes (LBRSSes) choose routes with longer expiration times and fewer disconnections. LBRSSes are appropriate for the cases with fewer active multicastings in the network. When the number of active multicastings increases, DBRSSes show better scalability and have comparatively fewer packet collisions and losses. Therefore, an appropriate route selection scheme is crucial in the design of multi-hop multimedia dissemination protocols due to that scheme’s impact on the packet losses (PLs) caused by disconnections, packet collisions, and low SNRs. This paper proposes a multicriteria adaptive opportunistic treecast routing protocol (MAOTRP) that adaptively selects an appropriate route according to the number of active multicastings and supports opportunistic packet forwarding to reduce PLs. Wireless mediums can share single transmissions with all nodes within the vicinity of the sender node, and the MAOTRP exploits this advantage. It supports opportunistic forwarding in the tree-based multicast scheme to provide robustness, as shown in Fig 1e, which reduces the average disconnection time (ADT). The route selection process in the MAOTRP considers lifetime and delay parameters (Fig 1c), and it adaptively selects their appropriate weights based on the number of active multicastings in the network. The varying number of multicastings effects the total network traffic, contention delays and packet collisions due to parallel multicast flows.

QoS refers to the transmission quality of a network, which mainly depends on the network parameters such as the packet loss ratio (PLR), jitters, delays, bandwidth, and burstiness. In fact, QoE also strongly depends on the network QoS parameters [6]. By considering network latency, lifetime and opportunistic packet forwarding, our proposed scheme improves the network QoS and user QoE. We organize the rest of the paper as follows. Section 2 presents the background and related work. We explain the VVT network model and assumptions in Section 3. Section 4 explains the proposed MAOTRP in VVT, and Section 5 provides the evaluation results. We conclude the paper in Section 6.

## 2 Background and related work

Digital signage (DS) displays advertisements and infotainment services in public places. Declining hardware prices, context awareness, and the shift toward greater interactivity are the main factors driving DS services. DS system typically distributes advertisements, TV broadcasting, and government announcements to trains, buses, transport terminals and hospitals [7]. A recent study [8] proposed a quality of service (QoS) and mobility-aware in-vehicle telescreen that extended the DS framework [9] to vehicular networks by connecting the first hop neighbors of the roadside unit (RSU) to the DS.

In the last decade, researchers have proposed different schemes for multimedia content delivery in VANETs. The AdTorrent system [10] supports push-based content delivery from a roadside infrastructure node (called a digital billboard) to first hop neighbors. CarTorrent [11] is a P2P file sharing protocol in VANETs that uses an AODV routing algorithm for data dissemination. P2P clients may join or depart the network frequently, and the routing information in the P2P clients may become stale, leading to lookup failures [12]. Reconfiguring the P2P overlay network increases the overhead under a dynamic VANET environment. Eugster et al. [13] proposed a diffusion process using an epidemic algorithm in which nodes relay received information to randomly selected nodes. The random selection of nodes in the epidemic algorithms may lead to higher delays. Jarupan et al. [14] proposed a position-based routing protocol for V2I communication which used the direction information contained in the packets. The routing information in the packets may become stale due to dynamic environment. Consensus protocols [15–17] have been applied for trusted data dissemination in vehicular adhoc networks. Distributed and global consensus protocols can dynamically adopt the consensus value among the service subscribers to optimize the consensus objective functions.

Most multi-hop multimedia dissemination schemes use controlled-broadcasting protocols [18–20] to minimize redundant transmissions. These broadcast-based schemes deliver content to all nodes in a target area, but subscription-based services are intended only for service subscribers to whom content can be efficiently delivered using multicast routing protocols. Santamaria et al. [21] proposed a partitioned multicast tree protocol (PAMTree) that considered the signal interference-to-noise ratio (SINR) and link consistency metrics as path selection criteria. In PAMTree, the source selects a route to each group member which may not be appropriate in dynamic topologies. An improved multicast ad hoc on-demand distance vector (MAODV) protocol considered the network load from the RSU to the destination node as path selection criteria [22]. Another research study focused on the adjustment of the transmission rate and the power level of the physical layer to minimize PLs due to interference [23]. A lot of research has applied opportunistic multicast routing (OMR) schemes for content distribution in VANETs [24, 25]. These OMR schemes take advantage of broadcast overhearing but add delays in relaying packets at each hop.

Encoded video streams are either composed of intra-coded (I), predictive-coded (P) and bidirectionally predictive-coded (B) frames, or they are composed of I and hierarchical B-frames in a group of pictures (GoPs) with multiple enhancement layers (ELs). These encoded frames have different degrees of importance on the user QoE [26]. Depending on the position of a lost packet at a specific EL, some of the frames in the higher ELs are also affected and not decodable. For evaluation of the perceived visual quality, many metrics proposed in the literature can be used such as video quality metric (VQM), Peak Signal to Noise Ratio (PSNR), Structural Similarity (SSIM), and Mean Opinion Score (MOS).

In this paper, we propose an adaptive multimedia dissemination protocol for VVT to extend DS service to VANETs. The main objective is to reduce the aggregate PLs, ADT, and delays. We propose an adaptive opportunistic treecast routing protocol that incorporates the efficiency of tree-based multicast schemes and the robustness of mesh-based multicast schemes. The proposed route selection scheme adaptively selects the weights of the route selection parameters by considering their contribution in improving the packet delivery ratios (PDRs). Through extensive simulation via the Omnetpp [27], Veins [28], and SUMO [29] tools with video traces from the Video Trace Library [26], we prove the effectiveness of the proposed scheme regarding ADT, network QoS, and user QoE.

## 3 Vehicle-to-Vehicle telescreen network model and assumptions

**Fig 2:**
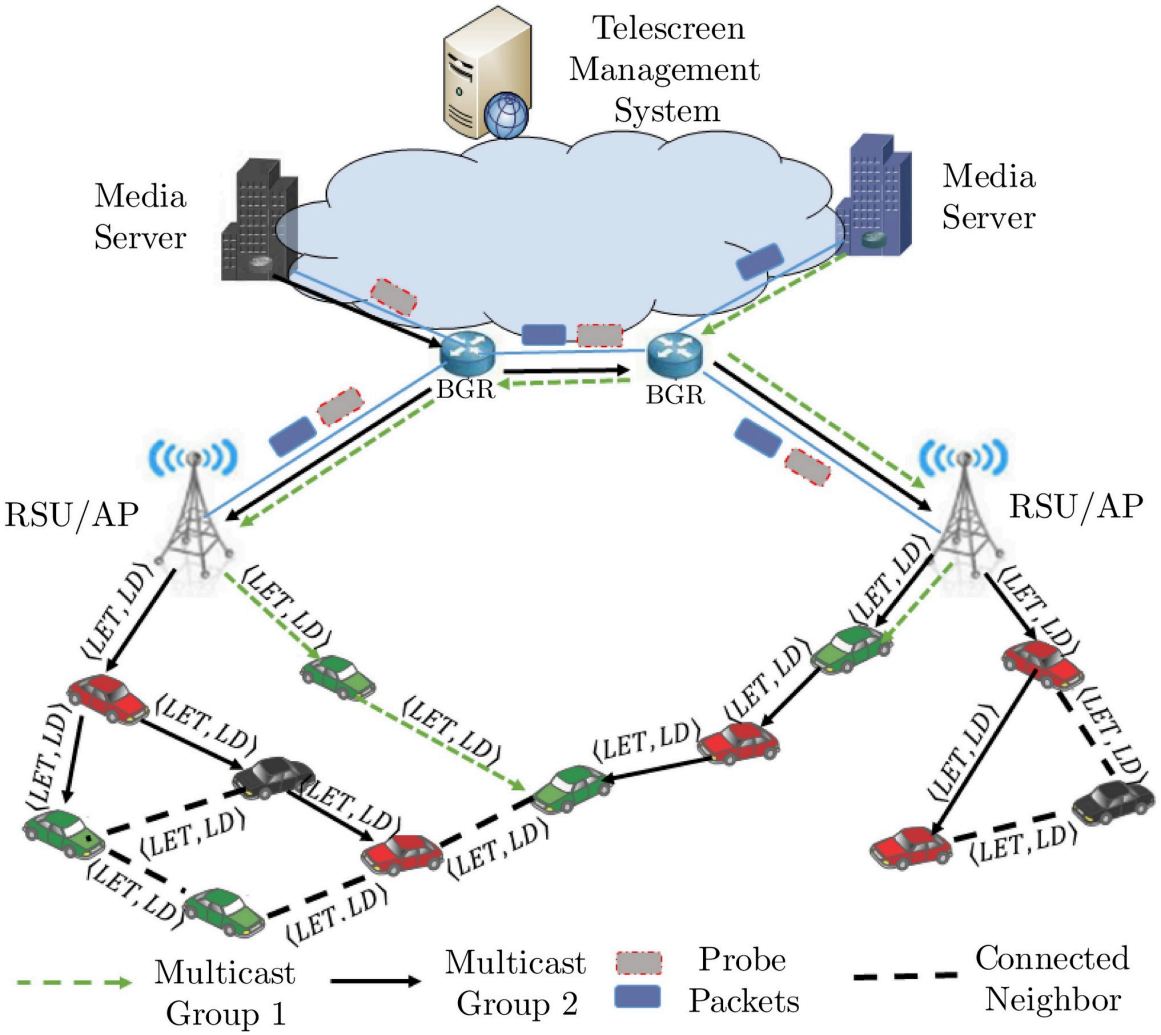
Vehicle-to-vehicle telescreen network model.

This section explains the VVT network model, notations, and assumptions. VVT extends the high-resolution and delay-sensitive DS services in VANETs, as shown in Fig 2. VVT can provide infotainment services from different service providers. VVT includes the logical and functional nodes of the telescreen management system (TMS), media server (MS), RSU, and vehicle. The TMS is the central repository for vehicle and service registrations and advertisements. The RSU works as a service gateway between the DS system and the VANET. A vehicle can either play the role of a service subscriber or a forwarding node to support multi-hop dissemination. The MS delivers the requested streams to their subscribers in the VANET through the RSU. We consider the metrics of link expiration time (LET) and link delay (LD) on the edges, which helps in the selection of the appropriate route from an RSU to the vehicle. The link delay is composed of the queuing delay, processing delay, transmission delay, and propagation delay. The end-to-end delay of a route is the sum of delays of the links along the route. In this paper, we consider end-to-end delay parameter in the fit function of the route selection process. To find the network latency from the MS to an RSU, we use an existing probe packet train method [8].

In VVT, we consider *k* vehicles and assign a unique identifier (*i* ∈ [1, *k*]) to each of them. The combination of these nodes and their communication links can be represented as a graph *G*(*V*, *E*), where vertices *V* = {*v*_1_, *v*_2_, …, *v*_*k*_} denote the finite set of *k* nodes and *E* = {*e*_1_, *e*_2_, …, *e*_*n*_} represent the communication links between the nodes. We denote the set of first hop neighbors of node *v*_1_ as *N*(*v*_1_) ⊂ *V*, and the distance from sender node by *D*. We consider the following assumptions in VVT design.

1. All vehicles have an IEEE 802.11p compliant radio transceiver that enables both V2I and V2V communication types.
2. All nodes have a transmission range (R) of 300m.
3. All nodes have a GPS module for location awareness.
4. All nodes use the channel coordination specifications of the IEEE 1609-4 standard and the same service configurations.
5. Each node periodically broadcasts hello packets that include its location, direction, and speed information.
6. A node detects disconnection if it is at the border (*D* > 290*m*) and does not receive two consecutive packets of the subscribed telescreen multicast group (TMG).

## 4 The proposed multicriteria adaptive opportunistic treecast routing protocol

The main goal of this work is to efficiently support the dissemination of a delay-sensitive VVT service in a dynamic VANET environment. We explain the details of the proposed MAOTRP in this section.

### 4.1 Overview of the proposed MAOTRP in VVT

**Fig 3:**
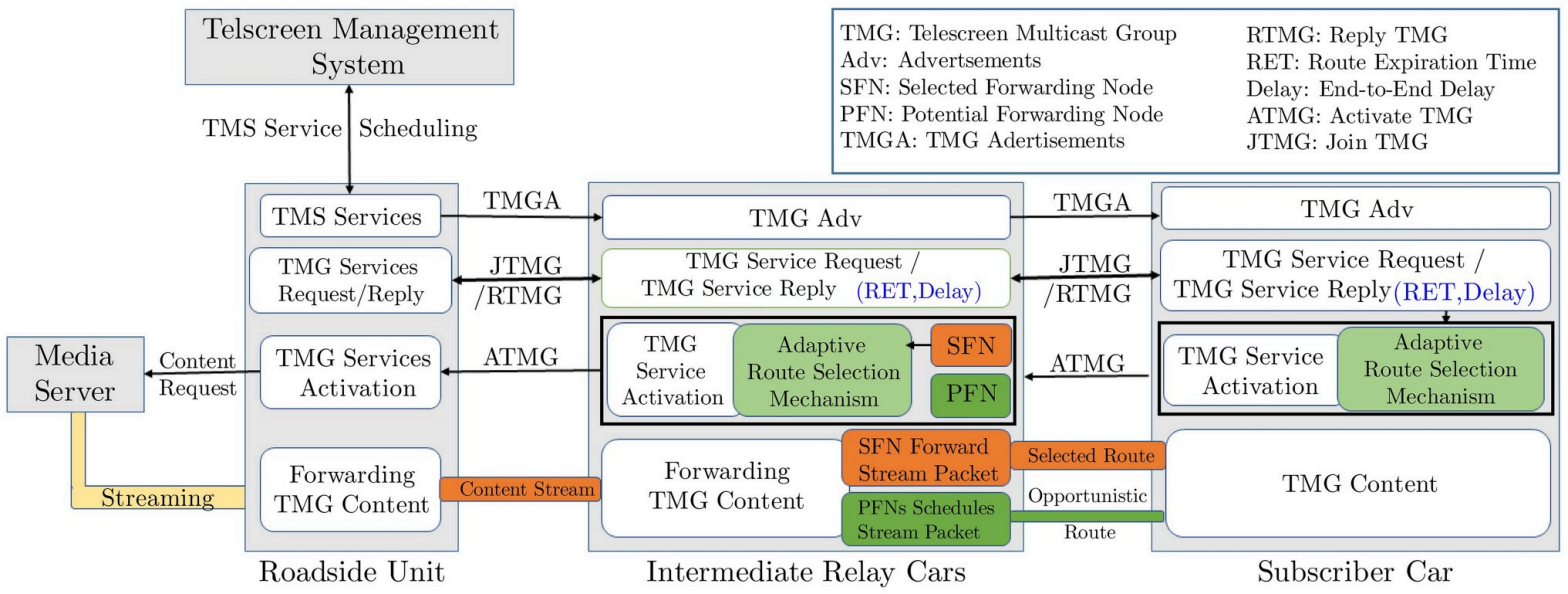
Overview of the proposed multicriteria adaptive opportunistic treecast routing protocol in vehicle-to-vehicle telescreen.

Fig 3 provides an overview of MAOTRP used to offer VVT service. According to service priority and location, the TMS schedules service advertisements with the target RSUs. The RSU periodically advertises the VVT services in its coverage area for subscriptions. A vehicle may join the advertised TMG by replying with the *join* TMG message. After receiving a subscription request, the RSU establishes connections with the MS (MS details are provided in the content’s meta-file) to acquire the content stream. The RSU broadcasts the TMG content stream which reaches all the subscribers through the proposed MAOTRP protocol detailed in the next subsection.

### 4.2 Multicriteria adaptive opportunistic treecast routing protocol

Multicast protocols commonly use messages to build and manage multicast trees to disseminate content from a source to the destinations. This subsection provides the details of the proposed multicast protocol for selecting the appropriate routes to destinations and the mechanism to select the potential forwarding nodes (PFNs) along the paths. Multicast tree nodes may fail to forward the incoming stream packets due to link disconnections, packet collisions or low SNRs. In such situations, one of the PFNs, at the hop where the failure occurs, relays the packets toward the downstream hops to reduce PLs. The MAOTRP extends the MAODV protocol. We exploit the wave short message protocol (WSMP) in building and managing the proposed MAOTRP. The MAOTRP protocol consists of advertisement, join, reply, activation, TMG dissemination, and maintenance phases as follows.

#### 4.2.1 Telescreen multicast group advertisement (TMGA)

**Fig 4:**
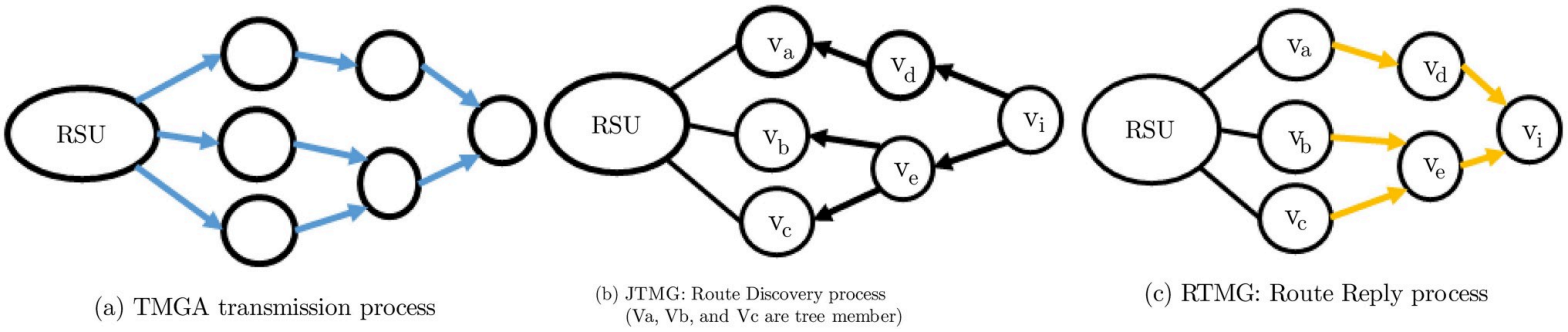
Overview of the dissemination of the TMGA, JTMG and RTMG messages.

**Fig 5:**
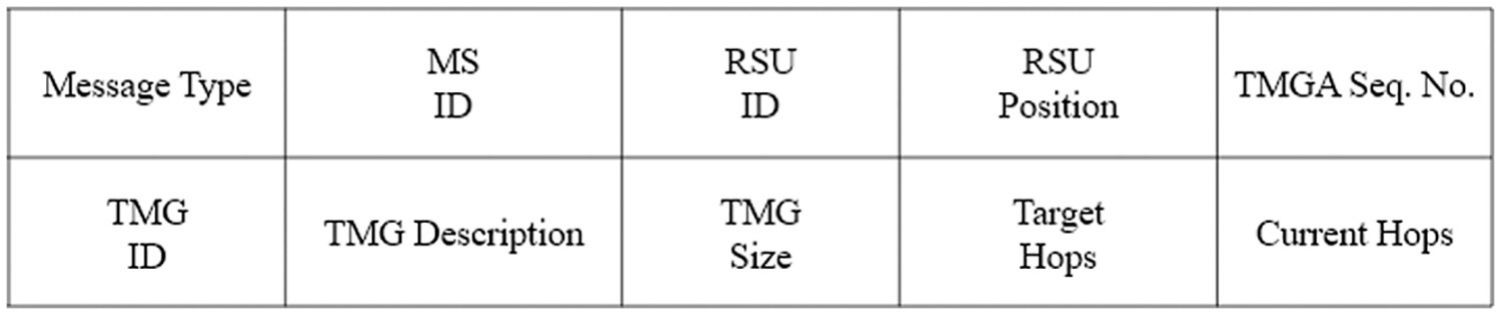
Portion of the TMGA packet.

**Fig 6:**
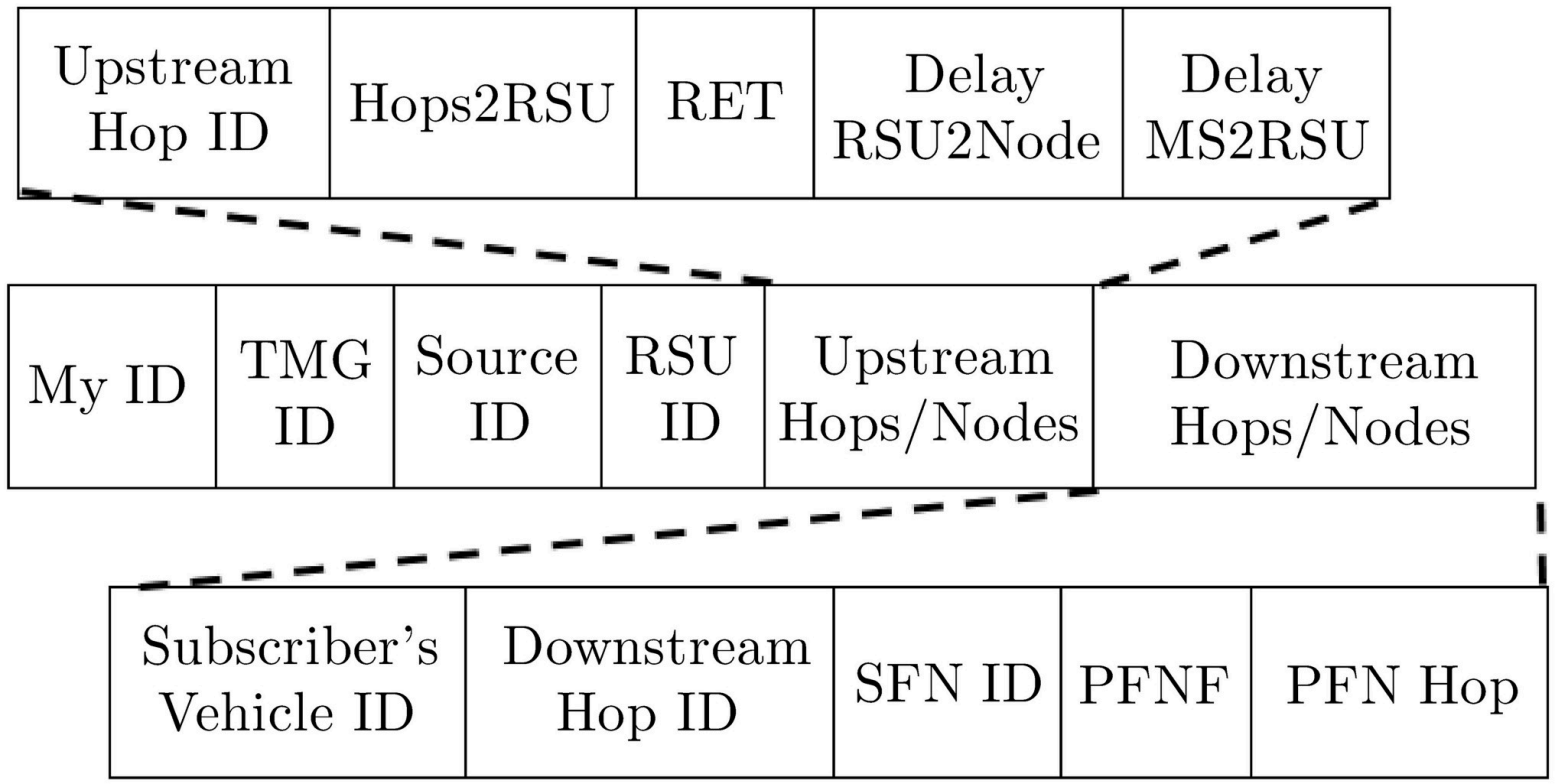
VVT services and subscribers table.

The RSU periodically broadcasts the TMGA to advertise the VVT video content scheduled by the TMS in the wave service advertisement (WSA) message of the WSMP. Fig 4a illustrates the multi-hop dissemination process, and Fig 5 shows the template of the TMGA message. Each node broadcasts the TMGA message only once until it reaches the target number of hops. Upon receipt of the TMGA message, nodes add the service information and the upstream hop information to the services and subscribers table (SSTable), as depicted in Fig 6. The route expiration time (RET) and delay fields are set as undefined in the SSTable during the advertisement phase.

#### 4.2.2 Join telescreen multicast group (JTMG)

**Fig 7:**
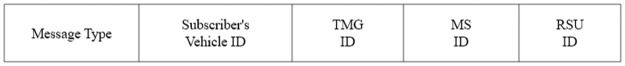
Portion of the JTMG packet.

A node can subscribe to the advertised TMG service by replying with a JTMG message. Each intermediate node updates the information from the downstream hops in the SSTable for the requested service and subscriber and broadcasts the request only once toward the RSU if it is not a member of the TMG. The selected forwarding node (SFN), potential forwarding node (PFN), and PFN at hop (PFN Hop) fields of the SSTable for the service and the subscriber are set as undefined during the join phase as these fields are collected later during the activation phase. Fig 4b shows the multi-hop dissemination process, and Fig 7 provides the template of the JMGA message.

#### 4.2.3 Reply telescreen multicast group (RTMG)

**Fig 8:**
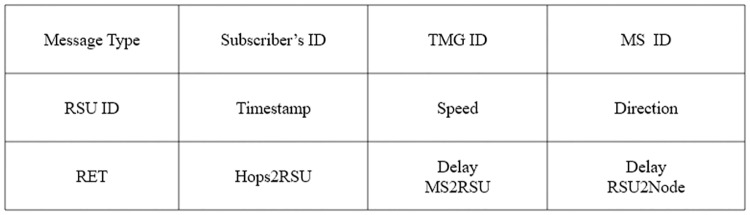
Portion of the RTMG packet.

For appropriate route selection, the RTMG message should provide values of the decision metrics such as the lifetime, delays, and number of hops to the subscriber. Either the RSU or a member of the TMG can reply to the JTMG, which follows the reverse path to the subscriber node, as shown in Fig 4c. Upon receipt of the RTMG message, nodes update the upstream hops information in the SSTable by calculating the RET and delay from the RSU to the node. We approximate the delay from the RSU to the node by subtracting the packet_sent_time (timestamp in the RTMG message) from its receipt time, which incorporates all of the intermediate processing, queuing, transmission, and propagation delays. The delay from the MS to RSU is constant along all paths in the VANET and is obtained from the RTMG message of the RSU. The intermediate relay nodes also update all of the fields including the RET, delays, and the number of hops to the RSU, in the RTMG message, as shown in the Fig 8. The RET is equal to the minimum LET along the path. The RET at node *v*_*i*_ is the minimum value between the RET of the previous relay node (*v*_*i*−1_) to the RSU and the LET between node *v*_*i*_ and the *v*_*i*−1_, as given by

$$\begin{array}{c} RET\left(v_{i}\right)=\min\left(RET\left(v_{i-1}\right),LET\left(v_{i},v_{i-1}\right)\right). \end{array}$$ (1)

To estimate the LET, let (*x*_*i*_, *y*_*i*_) and (*x*_*r*_, *y*_*r*_) denote the GPS coordinates of vehicles *i* and *r*. Knowing the speed *v* and direction *θ* (such that 0 ≤ *θ* < 2*π*) of vehicles *i* and *r*, and their transmission range *R* = 300*m*; we can estimate the *LET*(*i*, *r*) [30] as

$$\begin{array}{c} LET\left(i,r\right)=\frac{(\sqrt{\left(a^{2}+c^{2}\right)R^{2}-{\left(ad-bc\right)}^{2}})-ab-cd}{a^{2}+c^{2}}, \end{array}$$ (2)

where

$$\begin{array}{c} \{\begin{array}{c} a=v_{i}*cos\left(\theta_{i}\right)-v_{r}*cos\left(\theta_{r}\right),\\ b=x_{i}-x_{r},\\ c=v*sin\left(\theta_{i}\right)-v_{r}*sin\left(\theta_{r}\right),\\ d=y_{i}-y_{r}. \end{array} \end{array}$$

#### 4.2.4 Activate telescreen multicast group (ATMG)

Frequent route disconnections due to high-speed mobility in the VANET lead to an increase in PLs and, as a result, degrade the PDR. Selecting a stable route with the minimum number of disconnections may also increase the number of hops to the RSU, thereby causing delays. Multimedia services have stringent delay requirements, and a packet which exceeds its maximum delay limit is considered lost. According to [30], LET depends on the position, speed, transmission range, and direction of the link’s nodes. Due to neighbors’ contention and queuing delays, only a portion in an average of LET of the considered link can be available for transmission. Consequently, the RET/LET alone is a less effective parameter in the design of multimedia dissemination protocol for VANETs. Neighbors’ contention affects the transmission delay [31, 32]. To compensate the effect of contention and queuing delays to the considered link, we incorporate both RET and delay parameters in the design of MAOTRP using the following fit function (*FF*)

$$\begin{array}{c} FF=\alpha *\frac{RET(v_{i})}{A}+\frac{1-\alpha }{T_{D}*TotalPackets_{RET}}, \end{array}$$ (3)

where

$$\begin{array}{c} \left\{ \begin{array}{c} Alpha=\alpha =\frac{1}{1+(.1*{\left(.6325\right)}^{-ActM})},\\ 0<\alpha <1,\\ ActM=Number\;of\;Active\;Multicastings,\\ T_{D}=D_{MS2RSU}+D_{RSU2Sub},\\ D_{MS2RSU}=Delay\;from\;Media\;Server\;to\;RSU,\\ D_{RSU2Sub}=Delay\;from\;RSU\;to\;Subscriber,\\ TotalPackets_{RET}=RET*Frame\;Rate,\\ A=Normalization\;Constant\;of\;the\;RET=6. \end{array}\right. \end{array}$$

**Fig 9:**
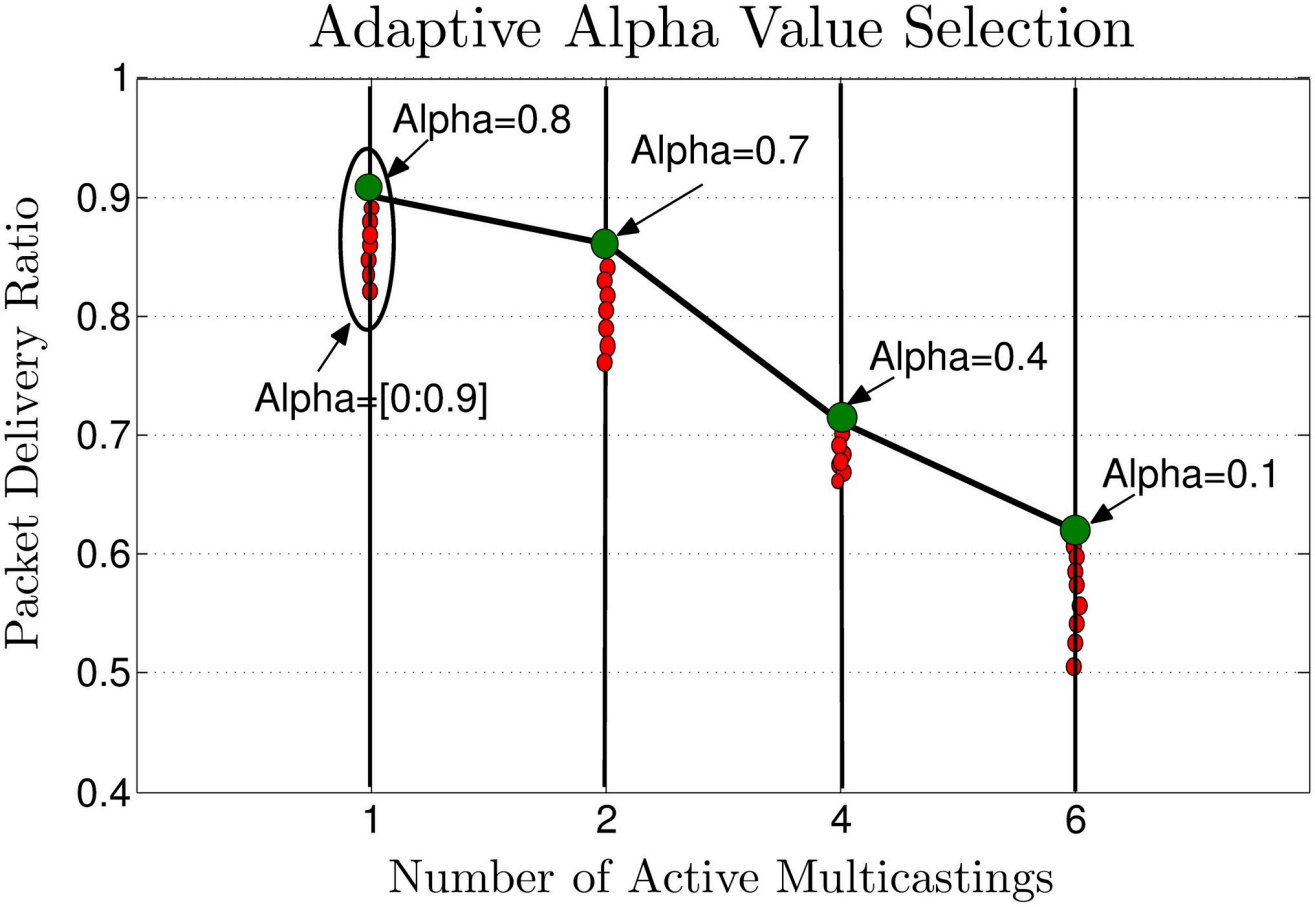
Adaptive parameter’s weight selection.

**Fig 10:**
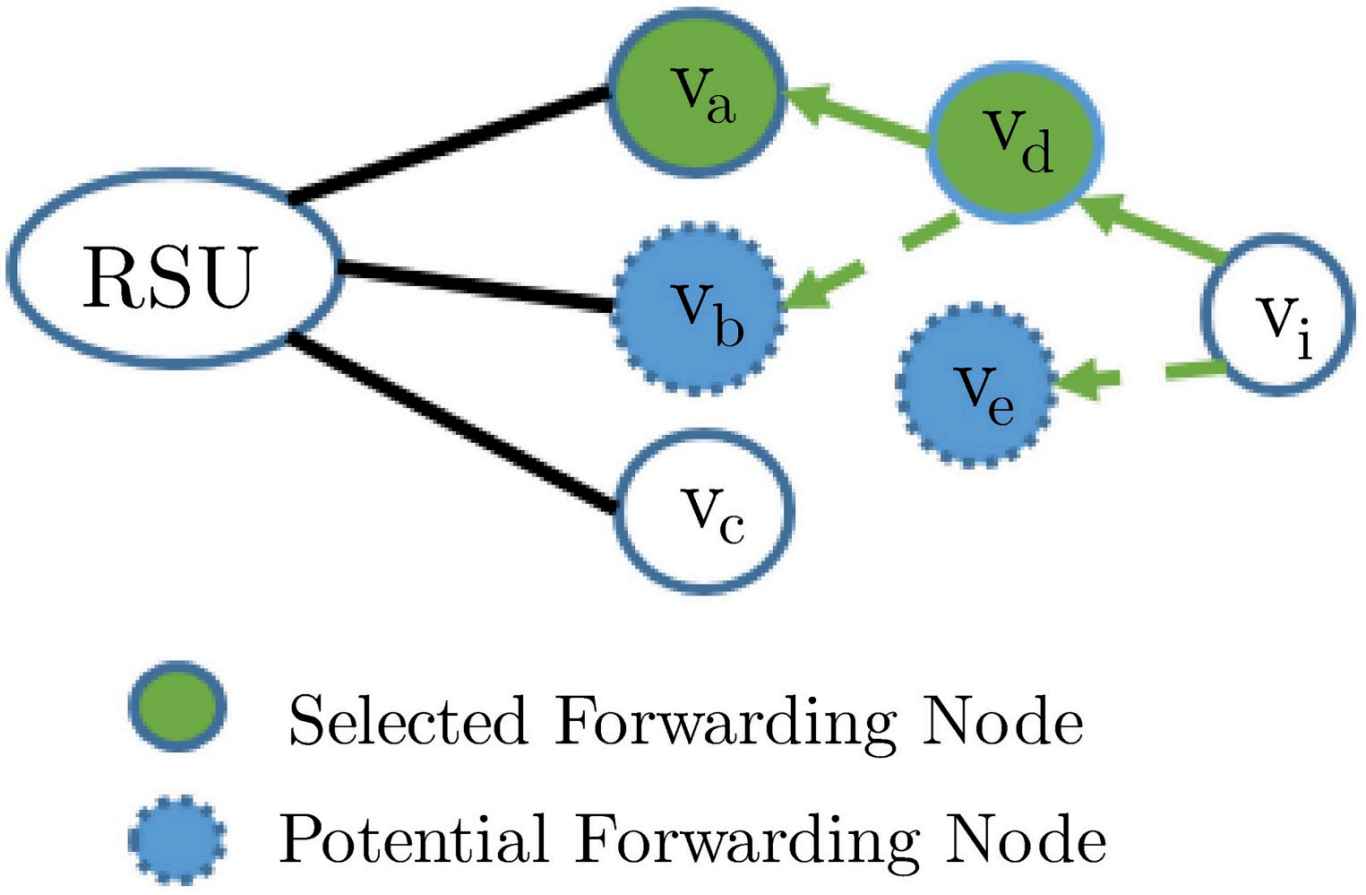
ATMG message dissemination.

The *FF* considers the route lifetime and the approximated total delay to be experienced by the stream’s packets during that lifetime. Parameter alpha, *α*, gives relative importance to the lifetime and delay parameters by considering their contribution in improving the PDR. The appropriate value of alpha depends on the number of active multicastings, number of users in the multicast groups, vehicle speed and vehicle density. Due to non availability of datasets and to reduce the simulation time, we only consider the number of active multicastings to decide the importance of Lifetime and Delay parameters in the route selection process in this paper. Through simulations, we find the appropriate value of *α* to reduces the PLs, as depicted in Fig 9. We use curve fitting to approximate the equation of *α* with an inverse logistic function. A subscriber or the intermediate relay selects the upstream hop, say *X*, whose route gives the maximum value for the *FF*. Upon receipt of the ATMG message, node *X* updates the SSTable and sets itself as the SFN for the downstream hop and/or subscriber. Another intermediate node, *v*_*i*_, may set its PFN flag and PFN Hop field of the SSTable if both SFN ‘X’ and the subscriber/downstream hop belong to *N*(*v*_*i*_). Fig 10 demonstrates the dissemination of the ATMG message, the selection of the SFN, and the PFN nodes along the path.

#### 4.2.5 TMG dissemination

After receiving the ATMG message, the RSU or the selected TMG member starts forwarding/broadcasting the incoming TMG packets to the downstream hops. Upon receipt of a TMG packet, the SFN forwards it without incurring any additional delay. However, each of the PFNs (if any exist) starts a timer to broadcast the packet if it does not overhear the packet from the SFN or another PFN during the ‘timer’ period. The PFN sets the timer as

$$\begin{array}{c} Timer=MaxWaitTime*(1-\frac{D}{R}), \end{array}$$ (4)

where *D* is the distance between the sender and current node, and *R* is the transmission range. The *MaxWaitTime* is an adjustable configuration parameter and we set it as 0.01s.

#### 4.2.6 Route maintenance and mobility management

Route failure occurs if one of the intermediate route’s links breaks. When a subscriber detects mobility, the link to its upstream SFN breaks, it sends a JTMG request with a different sequence number to repair the route. Upon receipt of the new JTMG from the subscriber, nodes set the SFN, PFN and PFN Hop fields for the subscriber and TMG as undefined in the SSTable. If an SFN detects mobility, it sets the SFN ID field as undefined in the SSTable and sends a TMGError message to the downstream hops. Upon receipt of the TMGError, the potential forwarding nodes set the PFN and the PFN Hop fields of the SSTable as undefined. Similarly, all of the intermediate SFNs set the SFN ID as undefined and forward the packet until it reaches all of the subscribers of the TMG who use the broken link. After receiving the TMGError message, the subscriber re-initiates the JTMG message to establish a route to the RSU with a different sequence number.

While moving along the road, a node may receive TMGAs from multiple RSUs with a different number of hops. If the hops to a new RSU are less than the hops of the current route, the subscriber sends a JTMG to the RSU to collect the lifetime and delay information of the available routes. If the FF value of a path to the investigated RSU is greater than the FF value of the current route, the subscriber sends an ATMG message to the new RSU and sends a LeaveTMG message to the previous RSU.

## 5 Simulation results

In this section, we study the performance of the proposed MAOTRP and its comparison with the existing MAODV [3] and Motion MAODV (MMAODV) [4] protocols. For comparison purpose, we implement the MAODV protocol with both the delay-based (D-MAODV) and Hop-based (H-MAODV) route selection mechanisms.

### 5.1 Simulation setup

We perform simulations to evaluate the performance of the proposed protocol in the Omnetpp network simulator [27]. We use the WAVE protocols stack implemented in the Omnetpp veins framework [28]. We import real maps from OpenStreetMap [34] to the SUMO tool [29], allowing us to generate the desired vehicles flow. The TraCI module, implemented in the veins framework, couples the Omnetpp and SUMO tools to work together. Table 1 shows the main parameters used in the simulations. We assign the messages of the protocols to different channels of the 802.11p standard, as shown in Table 2. We use the publicly available online video traces at the Video Trace Library [26] in these simulations. We transmit the same video trace in all of the active multicastings.

**Table 1:** Simulation parameters.

Parameter	Description
Highway length	3000m
Simulation Time	100 sec
Wireless interface	IEEE 802.11p
Channel Bandwidth	6 Mbps
useServiceChannel	true
phy80211p.sensitivity	-94 dBm
phy80211p.maxTXPower	10mW
Propagation and Inference Model	TwoRayInterferenceModel [33]
usePropagationDelay	true
thermalNoise	-110 dBm
mobilityType	TraCIMobility
Transmission range	300 m
Subscribers per MG	2
Frame Rate	30
Advertisement Interval	2s
Hello packet Interval	3s
Vehicle Density	40, 60 veh/km
Vehicle Placement	Uniform distribution
vType maxSpeed	35, 100 m/s
Number of Active Multicastings	1,2,4,6

**Table 2:** Channel assignment.

Channel Name	Frequency	Assigned Messages
CCH	5.89GHz	ATMG, TMGError
SCH1	5.87GHz	TMGA, Content, Hello
SCH2	5.88GHz	RTMG
SCH3	5.90GHz	JTMG
SCH4	5.91GHz	LeaveTMG

### 5.2 Video Trace description

**Fig 11:**
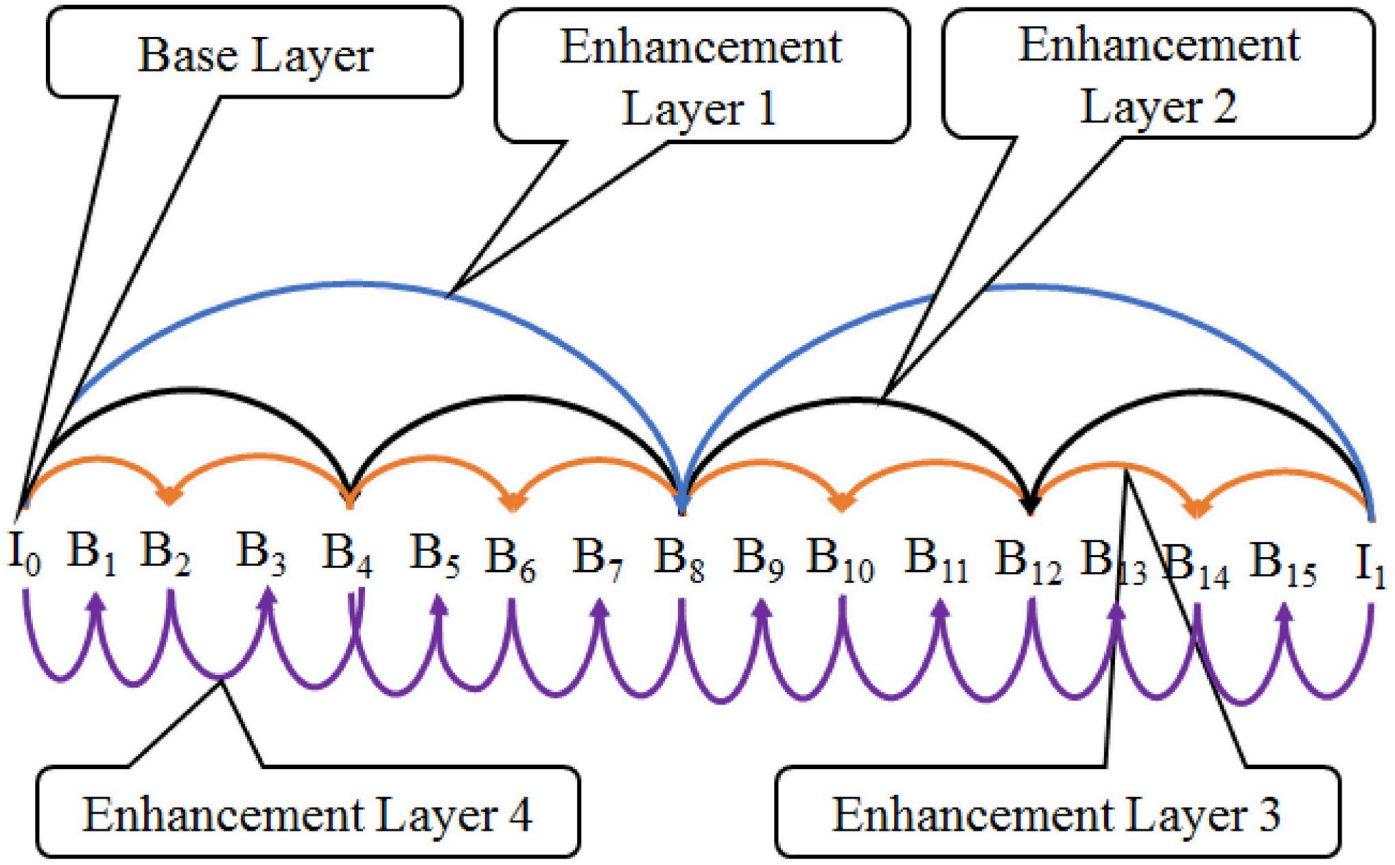
GoP structure with hierarchical B-frames.

In this subsection, we illustrate the video traces used in these simulations, which are publicly available online at the Video Trace Library [26]. Let’s consider the single layer SVC video sequence “Elephants Dream,” which has a GoP size of 16. Each GoP includes one I and 15 B-frames (Fig 11) with a BL and four ELs, as shown in Table 3. The table includes the frame number, playback time, frame type, frame size, and perceived visual quality values of the luminance and two chrominance components. Suppose that the packet carrying Frame#212 is dropped due to a collision or low SNR. Considering the GoP structure (Fig 11), all of the frames from Frame#209 to Frame#215 are dependent on the dropped frame and cannot be decoded. Using an error concealment mechanism that redisplays the last successfully received frame for the lost frames (or frames that cannot be decoded), Frame#208 is redisplayed. We can acquire the visual quality, measured through the PSNR metric, of the lost frames from the offset distortion trace of Frame#208 which is available at the referred trace library. For instance, the PSNR value of Frame#209 is equal to the PSNR at Offset = 1 of the distortion trace of Frame#208.

**Table 3:** Trace description: “Elephants Dream Video Trace”.

Frame	Time	Type	Size	PSNR	PSNR	PSNR
#			Bytes	Y	U	V
208	8.66	I	3322	39.8	42.9	46.4
209	8.70	B1	64	39.6	42.9	46.2
210	8.75	B2	105	39.4	42.9	46.2
211	8.79	B3	79	39.1	42.9	46.4
212	8.83	B4	293	39.1	43.2	46.4
213	8.87	B5	81	38.9	43.1	46.2
214	8.91	B6	133	39	43.0	46.2
215	8.95	B7	84	38.9	43.1	46.4
216	9	B8	705	39.1	43.3	46.5

### 5.3 Evaluation and comparison

**Fig 12:**
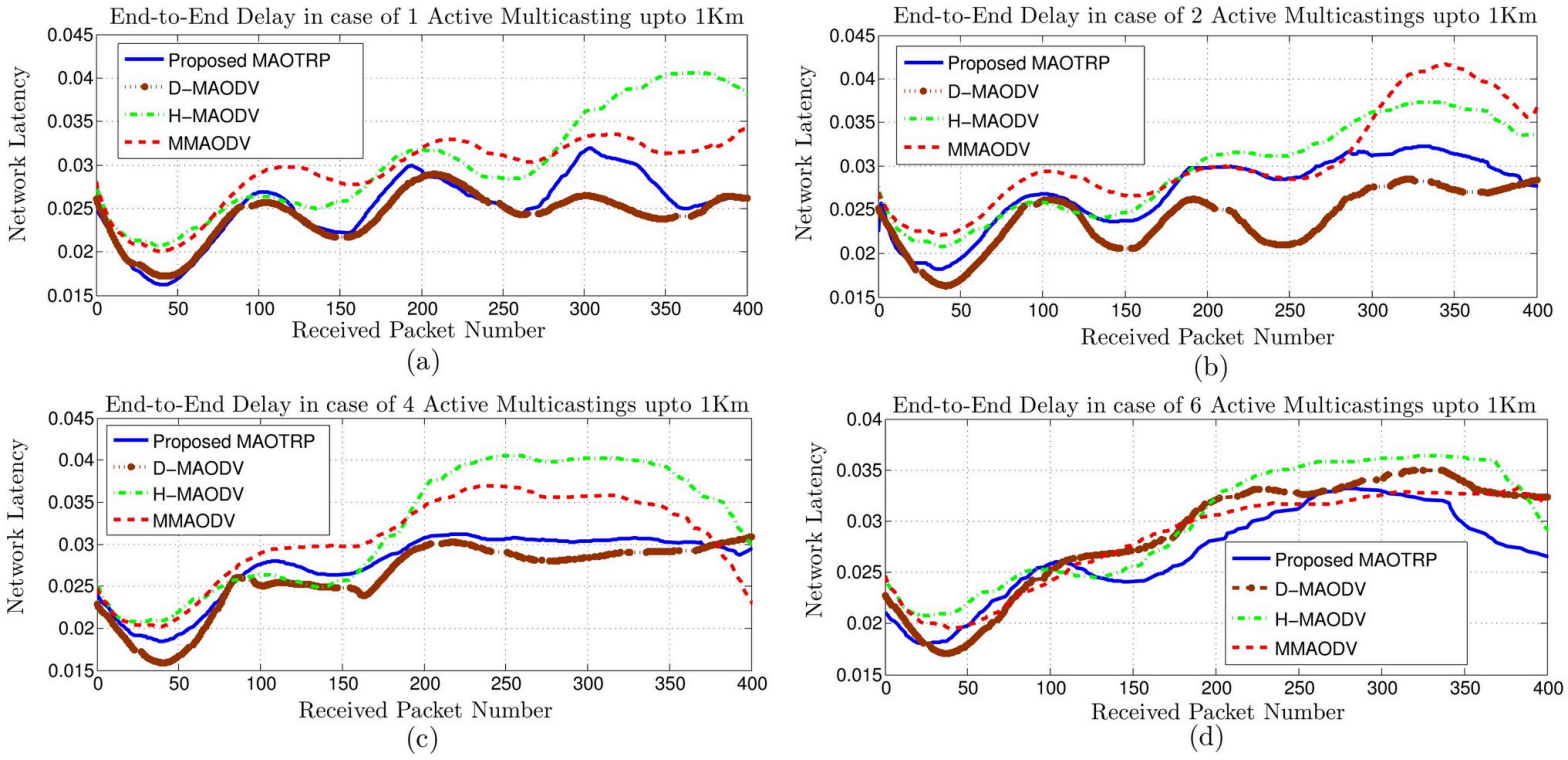
Impact of route’s lifetime and delay parameters on the network latency and disconnections.

We compare the performance of the proposed MAOTRP with the existing schemes in terms of network latency, PDR, average application throughput, ADT and user QoE. For all of these results, we plot the data collected at the reference node, *’Node [0]’*, which is a member of TMG 1 and is the farthest node from the RSU. We conduct a set of simulations with different densities (vehicle per kilometer), vehicle speeds, and numbers of active multicastings to check the applicability of the MAOTRP in various environments. We show the comparison of the schemes in a network configuration with a density of 40 veh/km and maximum vehicle speed of 100 m/s. Fig 12 shows the impact of the weights of lifetime and delay parameters on the number of disconnections and network latency. For this simulation, we turn off the multichannel operation of the 802.11p and consider only one multicasting to clearly visualize the delays, disconnections, and impact of the opportunistic packet forwarding scheme. In this simulation setup, giving more importance to lifetime (Case2) reduces the number of disconnections by 33% and improves the PDR by 5.5%. However, Case2 suffers 8.2% more from end-to-end delays than Case1. The average hop distance in Case1 and Case2 is 3.5 and 5 respectively. The spikes in the figure represent the receipt of packets during disconnections through the opportunistic nodes with extra timer’s delay.

**Fig 13:**
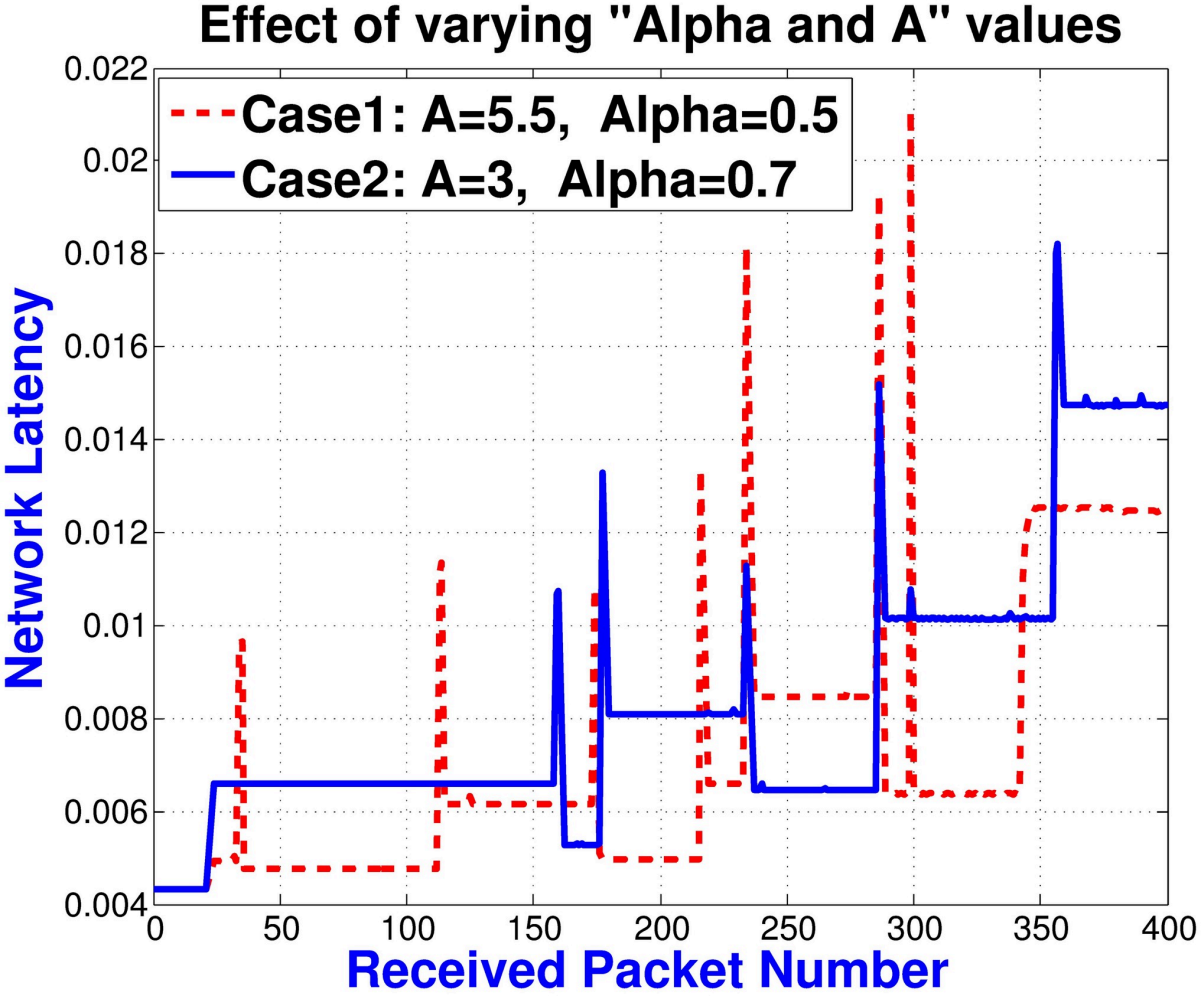
Efficiency in terms of network latency.

Fig 13 shows a comparison of MAOTRP with the existing schemes in terms of network latency. We adaptively select *α* according to the number of active multicastings. The proposed MAOTRP reduces the network latency by about 11.46% after 500m from the RSU than the average latency of existing schemes in these simulations. The waveform shape of network latency is due to averaging and the channel coordination mechanism of IEEE 1609-4 specifications. The source sends the stream’s packets at a constant interval. However, the link layer will queue the packets received from the upper layer if the service channel is not active.

**Fig 14:**
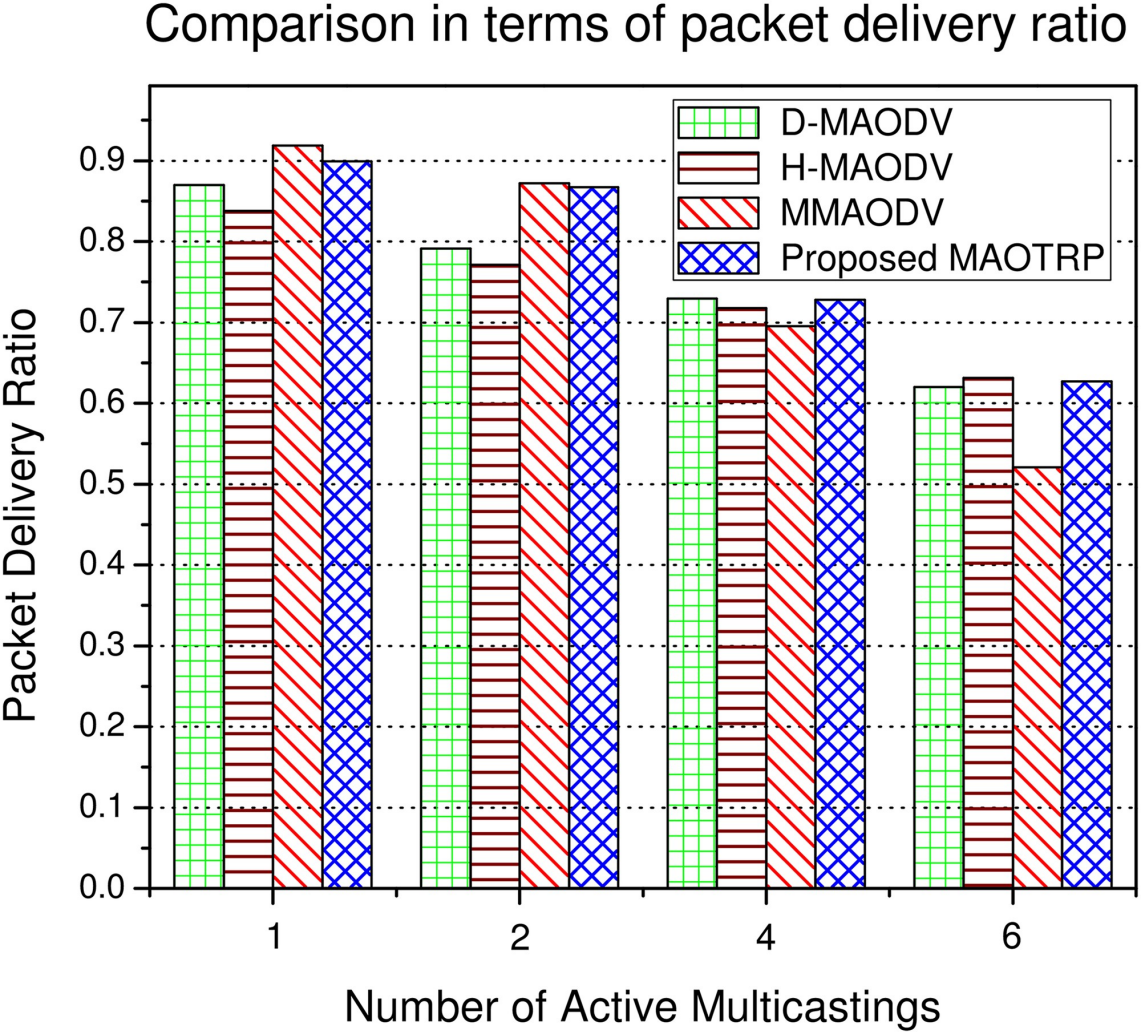
Efficiency in terms of packet delivery ratio.

The LBRSSes have a higher PDR than DBRSSes for a smaller number of active multicastings in the network, as depicted in Fig 14. However, LBRSSes have poor scalability and suffer more from PLs with an increase in the number of multicastings. This is because the selected routes are usually at a higher hop distance (the number of intermediate hops/links/relays) from the RSU, and they consequently experience more transmissions and collisions. The proposed MAOTRP adapts its route selection mechanism according to the active multicastings to consistently provide a high PDR. The MAOTRP improved the average PDR by about 4.21% in these simulations, as depicted in Fig 14.

**Fig 15:**
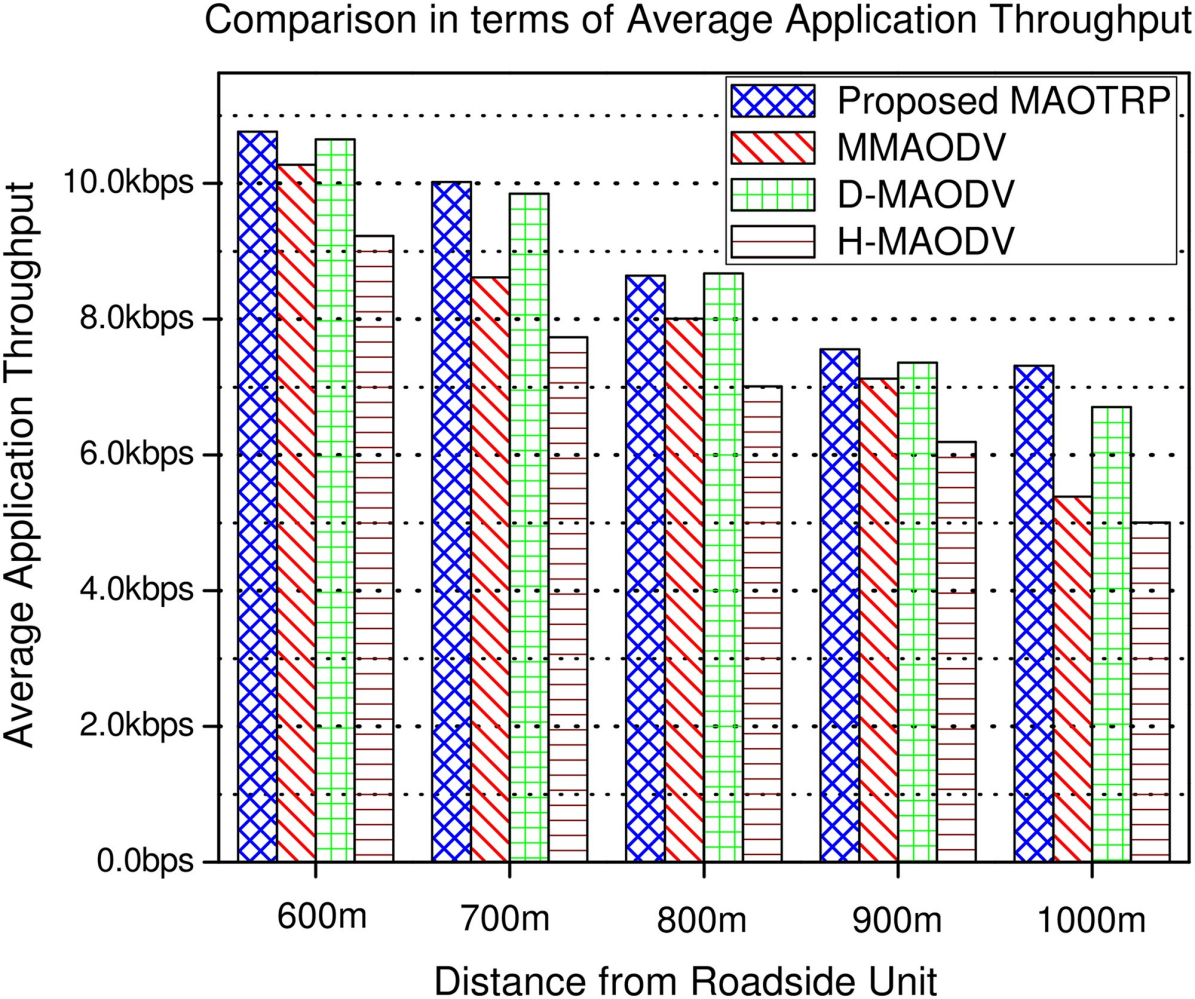
Efficiency in terms of average application throughput.

Fig 15 provides the comparison between the proposed and existing schemes in terms of application-level throughput, which is the number of useful bits received by the destination per unit of time. The simulations show that the proposed scheme improves the average application throughput by 13%. The average application throughput can be expressed as follows

$$\begin{array}{c} AAThr=\frac{1}{ActM}\sum_{j=1}^{ActM}\frac{1}{P_{j}}\sum_{i=1}^{P_{j}}\frac{PS_{i}}{RT_{i}-ST_{i}}, \end{array}$$ (5)

where,

$$\begin{array}{c} \left\{ \begin{array}{c} RT_{i}=Receipt\;time\;of\;the\;packet\;i,\\ ST_{i}=Sent\;Time\;of\;packet\;i,\\ ActM=number\;of\;active\;multicastings,\\ P_{j}=number\;of\;received\;packets\;for\;j\;ActM,\\ PS_{i}=size\;of\;the\;packet\;i. \end{array}\right. \end{array}$$

**Fig 16:**
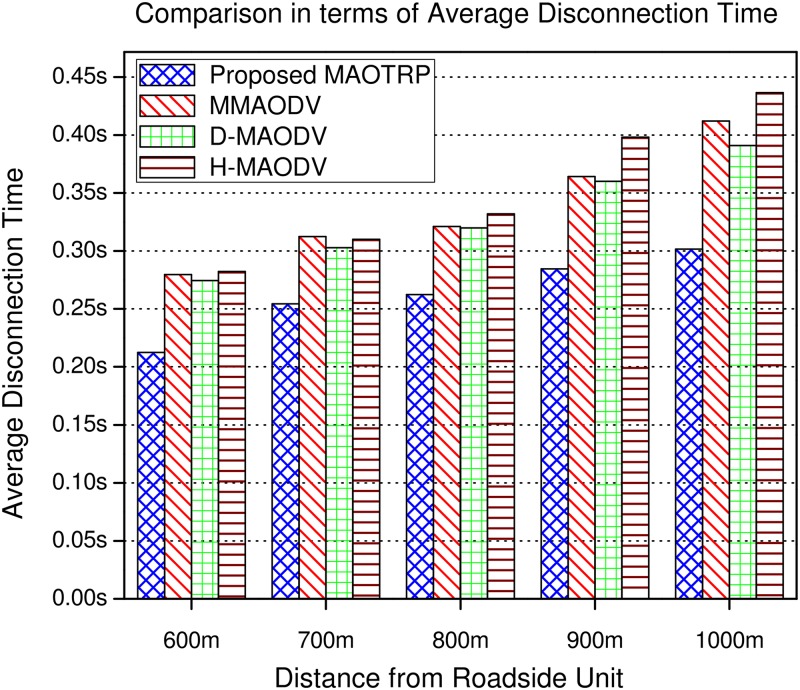
Efficiency in terms of average disconnection time.

During link disconnection, one of the potential relay node forwards the received packet to the downstream hops, which reduces the ADT and the number of consecutive dropped packets which impact user experience. The proposed MAOTRP protocol reduces the ADT by 22.75% than the existing schemes, depicted in Fig 16. We can express the ADT as follows

$$\begin{array}{c} ADT=\frac{1}{ActM}\sum_{j=1}^{ActM}\frac{1}{D_{j}}\sum_{i=1}^{D_{j}}\left(RT_{i}-ST_{i}\right), \end{array}$$ (6)

where,

$$\begin{array}{c} \left\{ \begin{array}{c} RT_{i}=Receipt\;time\;of\;the\;packet\;i\;at\;disconnection\;i,\\ ST_{i}=Sent\;Time\;of\;packet\;i\;at\;disconnection\;i,\\ ActM=number\;of\;active\;multicastings,\\ D_{j}=number\;of\;disconnections\;for\;j\;ActM. \end{array}\right. \end{array}$$

**Fig 17:**
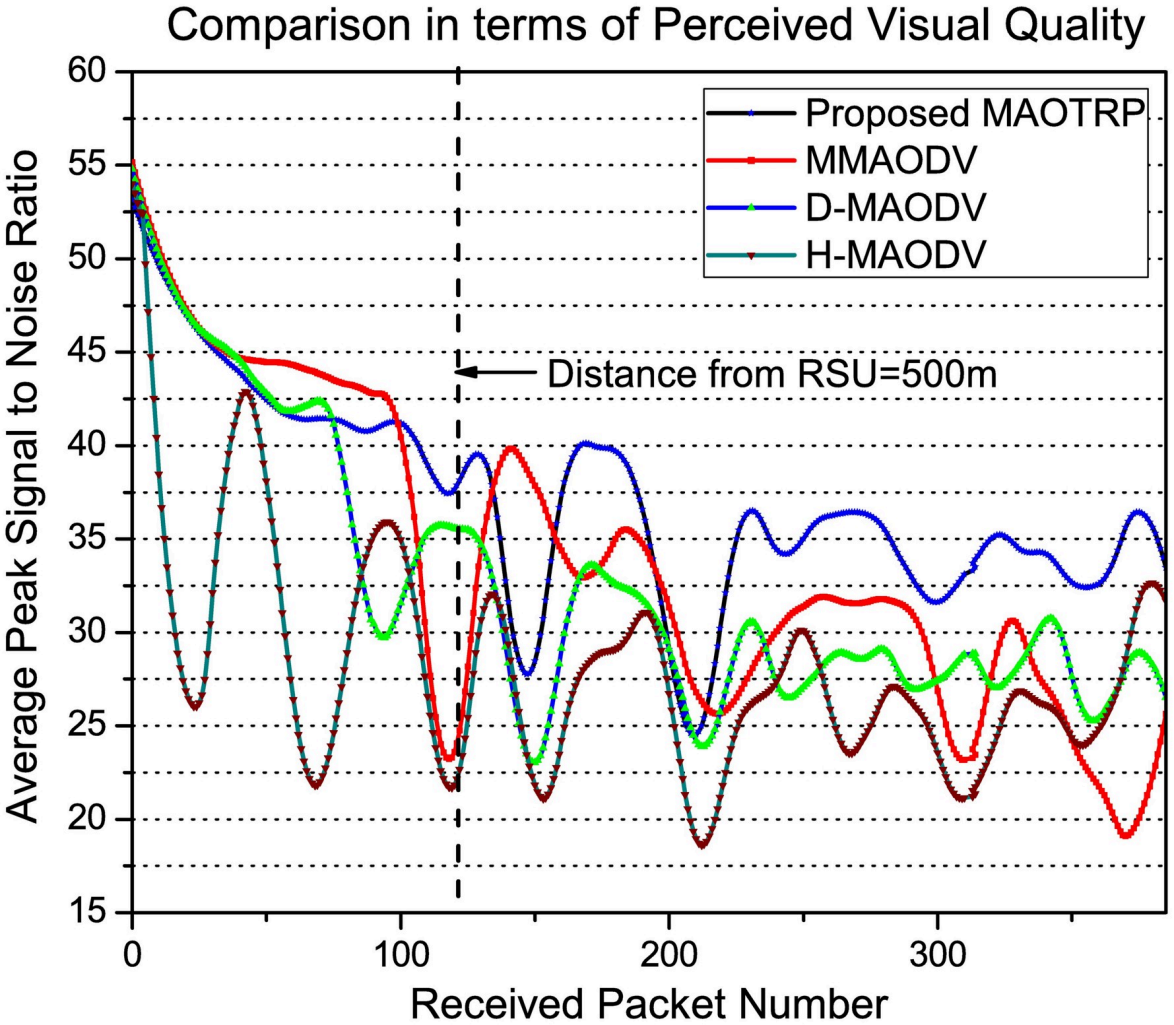
Efficiency in terms of user QoE.

**Fig 18:**
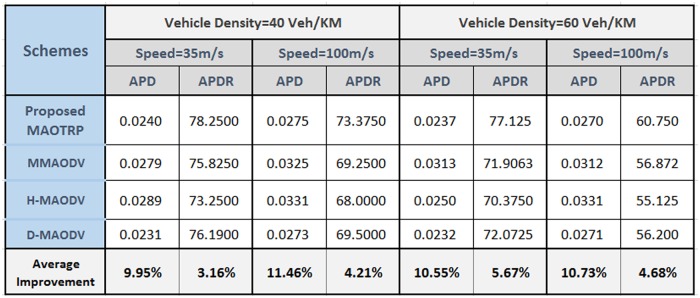
Comparison of the proposed scheme in terms of average packet delay (APD) and the average packet delivery ratio (APDR) of 1,2,4, and 6 active multicastings after 500 meters from the roadside unit in different simulation setups. Comparison of the proposed MAOTRP with the existing multimedia multicast schemes in different simulation setups.

As mentioned earlier, perceived visual quality strongly depends on the network QoS. By improving the application throughput and PDR as well as reducing latency and disconnection, the proposed MAOTRP also enhances the user experience. MAOTRP improves user experience by 6.16 dB after 500m from the RSU than the average of the existing compared schemes, as depicted in Fig 17. To check the applicability of the proposed in different environments, we vary the density and speed parameters in the vehicle’s flow. The proposed scheme performed better in terms of average packet delay and average PDR than that of the existing compared schemes in these simulations, as depicted in Fig 18.

## 6 Conclusion

In this paper, we have presented a multicriteria adaptive treecast routing protocol (MAOTRP) for vehicle-to-vehicle telescreen (VVT) designed to extend the infrastructure-based digital signage service to vehicular ad hoc networks (VANETs). This research aims to reduce packet losses (PLs), delays and disconnection time for enhancing the network QoS and user QoE. The proposed MAOTRP incorporates the efficiency of tree-based multicast schemes and the robustness of mesh-based multicast schemes. One of the potential relay nodes forwards the received multicast packet toward downstream hops in the case of tree’s link failure to reduce PLs caused by disconnections, packet collisions, and low signal quality. The proposed route selection scheme considers the fundamental trade-offs between the route lifetime and the delay parameters which exist due to the varying number of active multicasting in the VANET environment. It adapts the relative importance of the parameters according to their contribution in improving the packet delivery ratio (PDR). Through simulations and comparisons with existing schemes, on average, the proposed multimedia dissemination scheme in the VVT improves user QoE by 6.16 dB, average application throughput by 13% and PDR by 4.4%. Furthermore, compared to the existing schemes, MAOTRP reduces the average disconnection time by 22% and network latency by about 11% after 500m from the roadside unit.

## References

[1] NetWorld Alliance. The State of the Digital Signage Industry Survey; 2009.

[2] Bunn L. North America’s Digital Signage Industry: Status & Outlook; 2015.

[3] Royer E, Perkins C. Multicast ad hoc on-demand distance vector (MAODV) routing; RFC. Addison-Wesley.

[4] Jemaa IB, Shagdar O, Martinez FJ, Garrido P, Nashashibi F. Extended mobility management and routing protocols for internet-to-VANET multicasting; 2015 IEEE. pp. 904–909.

[5] Sinha P, Sivakumar R, Bharghavan V. MCEDAR: Multicast core-extraction distributed ad hoc routing; 1999 IEEE. pp. 1313–1317.

[6] Hyun Jong K, Seong Gon C. A study on a QoS/QoE correlation model for QoE evaluation on IPTV service,” in Advanced Communication Technology (ICACT). 2010. The 12th International Conference on, 2010, 1377–1382.

[7] Inoue H, Suzuki K, Sakata K, Maeda K. Development of a digital signage system for automatic collection and distribution of its content from the existing digital contents and its field trials; 2011 IEEE. pp. 463–468.

[8] Sarwar G, Ullah F, Lee H-W, Ryu W, Lee S. Quality of service and mobility aware in-vehicle telescreen service architecture; 2017 Tehnički vjesnik 24: 177–185.

[9] Ullah F, Sarwar G, Lee H-W, Ryu W, Lee S. Control framework and services scenarios of provisioning N-Screen services in interactive digital signage; 2014 Tehnicki Vjesnik-Technical Gazette 21: 1321–1329.

[10] Nandan A, Das S, Zhou B, Pau G, Gerla M. AdTorrent: Digital billboards for vehicular networks; 2005.

[11] Lee K, Yap I. CarTorrent: A bit-torrent system for vehicular ad-hoc networks; 2006 Los Angeles.

[12] Woungang I, Tseng F-H, Lin Y-H, Chou L-D, Chao H-C, Obaidat M-S. MR-Chord: Improved chord lookup performance in structured mobile p2p networks; 2015 IEEE Systems Journal 9: 743–751. 10.1109/JSYST.2014.2306147

[13] Eugster PT, Guerraoui R, Kermarrec A-M, Massoulié L. Epidemic information dissemination in distributed systems; 2004 Computer 37: 60–67. 10.1109/MC.2004.1297243

[14] Jarupan B, Ekici E. PROMPT: A cross-layer position-based communication protocol for delay-aware vehicular access networks; 2010 Ad Hoc Networks 8: 489–505. 10.1016/j.adhoc.2009.12.006

[15] Raghu Vamsi Krishna T, Barnwal R-P, Ghosh S-K. CAT: Consensus-assisted trust estimation of MDS-equipped collaborators in vehicular ad-hoc network; Vehicular Communications, 2(3), 150–157. 10.1016/j.vehcom.2015.06.001

[16] Shang Y. On the Delayed Scaled Consensus Problems; Applied Sciences, 7(7), 713 10.3390/app7070713

[17] Salvi A, Santini S, Valente A-S. Design, analysis and performance evaluation of a third order distributed protocol for platooning in the presence of time-varying delays and switching topologies; Transportation Research Part C: Emerging Technologies, 80, 360–383. 10.1016/j.trc.2017.04.013

[18] Cha S-H. A survey of broadcast protocols for vehicular ad-hoc networks; 2014 SmartCR 4: 246–255. 10.6029/smartcr.2014.04.001

[19] Yang J, Fei Z. Broadcasting with prediction and selective forwarding in vehicular networks; 2013 International journal of distributed sensor networks 9: 309041 10.1155/2013/309041

[20] Zhou L, Cui G, Liu H, Wu Z, Luo D. NPPB: A broadcast scheme in dense VANETs; 2010 Information Technology Journal 9: 247–256. 10.3923/itj.2010.247.256

[21] Santamaria AF, Sottile C, Fazio P. PAMTree: partitioned multicast tree protocol for efficient data dissemination in a VANET environment; 2015 International Journal of Distributed Sensor Networks. 10.1155/2015/431492

[22] Oe K, Koyama A, Barolli L. Proposal and Performance Evaluation of a Multicast Routing Protocol for Wireless Mesh Networks Based on Network Load; 2015 Mobile Information Systems 2015. 10.1155/2015/523294

[23] Kim J-S, Chung S-H. Adaptive on-demand multicast routing protocol for mobile ad hoc networks; 2015 International Journal of Distributed Sensor Networks. 10.1155/2015/652572

[24] Zeng G, Huang P, Mutka M, Xiao L, Torng E. Efficient opportunistic multicast via tree backbone for wireless mesh networks; 2011 IEEE. pp. 600–609.

[25] Chakchouk N. A survey on opportunistic routing in wireless communication networks; 2015 IEEE Communications Surveys & Tutorials 17: 2214–2241. 10.1109/COMST.2015.2411335

[26] Seeling P, Reisslein M. Video transport evaluation with H. 264 video traces; 2012 IEEE Communications Surveys & Tutorials 14: 1142–1165. http://trace.eas.asu.edu/, http://trace.kom.aau.dk/

[27] Varga A, Hornig R. An overview of the OMNeT++ simulation environment; 2008 ICST (Institute for Computer Sciences, Social-Informatics and Telecommunications Engineering). pp. 60 https://omnetpp.org/omnetpp

[28] Sommer C, German R, Dressler F. Bidirectionally coupled network and road traffic simulation for improved IVC analysis; 2011 IEEE Transactions on Mobile Computing 10: 3–15. http://veins.car2x.org/download/ 10.1109/TMC.2010.133

[29] Behrisch M, Bieker L, Erdmann J, Krajzewicz D. SUMO–simulation of urban mobility: an overview; 2011 ThinkMind. http://sumo.dlr.de/wiki/Downloads

[30] Benslimane A, Taleb T, Sivaraj R. Dynamic clustering-based adaptive mobile gateway management in integrated VANET—3G heterogeneous wireless networks; 2011 IEEE Journal on Selected Areas in Communications 29: 559–570.

[31] Li J, Chigan C. Delay-Aware Transmission Range Control for VANETs; in Global Telecommunications Conference (GLOBECOM 2010), 2010 IEEE, 2010, pp. 1–6.

[32] Taherkhani N, Pierre S. Improving dynamic and distributed congestion control in vehicular ad hoc networks; Ad Hoc Networks, vol. 33, pp. 112–125, 2015 10.1016/j.adhoc.2015.04.008

[33] Sommer C, Joerer S, Dressler F. On the applicability of Two-Ray path loss models for vehicular network simulation; 2012 14-16 11 2012 pp. 64–69.

[34] Haklay M, Weber P. Openstreetmap: User-generated street maps; 2008 IEEE Pervasive Computing 7: 12–18. 10.1109/MPRV.2008.80

